# The Prevalence of Single Nucleotide Polymorphisms of the *AOC1* Gene Associated with Diamine Oxidase (DAO) Enzyme Deficiency in Healthy Newborns: A Prospective Population-Based Cohort Study

**DOI:** 10.3390/genes16020141

**Published:** 2025-01-24

**Authors:** Emma Fortes Marin, Lydia Carrera Marcolin, Laia Martí Melero, María Tintoré Gazulla, Mireia Beltran Porres

**Affiliations:** 1 Unit of Neonatology and Pediatric ICU, Hospital Universitari General de Catalunya, C/Pedro i Pons 1, Sant Cugat del Vallés, 08195 Barcelona, Spain; mireia.beltran@quironsalud.es; 2 ABbiotek Health, 1 Samson Place, London Road, Peterborough PE7 8QJ, UK; lydia.carrera@abbiotekhealth.com (L.C.M.); laia.marti@abbiotekhealth.com (L.M.M.); maria.tintore@abbiotekhealth.com (M.T.G.)

**Keywords:** diamine oxidase enzyme, healthy newborn, *AOC1* gene, single nucleotide polymorphisms, genetic variants

## Abstract

**Background/Objectives:** The prevalence of the diamine oxidase (DAO) enzyme deficiency of a genetic origin has not been previously assessed. A prospective population-based study was conducted in a sample of 200 healthy newborns aimed to determine the prevalence of DAO enzyme deficiency caused by single nucleotide polymorphism (SNP) variants of the *AOC1* gene. **Methods:** Genotyping was performed in oral mucosa samples collected around 2 days after birth. The four more frequent SNPs, c.47C>T (rs10156191), c.995C>T (rs1049742), c.1990C>G (rs10449793), and c.691G>T (rs2052129), were analyzed. **Results:** DAO deficiency was present in 132 newborns, with a prevalence of 66% (95% confidence interval [CI] 59–73%). The rs10449793 variant showed a prevalence of 46%, followed by rs10156191 with a prevalence of 42.5%, and rs2052129 with a prevalence of 39.5%. The variant rs1049742 showed the lowest prevalence (9.5%). The frequency of one, two, three, or four SNPs was 23%, 23.5%, 10.5%, and 9%, respectively. In all fours SNP variants, heterozygous carriers were more frequent than homozygous carriers (19% homozygosity). Differences in the prevalence of DAO deficiency between males (68%, 66/96) and females (63.4%, 66/104) were not found (*p* = 0.885). The prevalence in Caucasian newborns was 66.5% (123/185), as compared with 60% (9/15) in Latin Americans (*p* = 0.821). **Conclusions:** This study carried out in healthy newborns indicates that there is a high prevalence (66%) of DAO deficiency of a genetic origin in the general population.

## 1. Introduction

The amine oxidase copper-containing 1 (*AOC1*) gene located in chromosome 7 (7q34-q36) encodes for the diamine oxidase (DAO) enzyme [1]. DAO is a secretory protein stored in plasma membrane-associated vesicular structures in intestinal epithelial cells and responsible for the degradation of extracellular histamine [2,3]. DAO inhibits the transepithelial permeation of exogeneous histamine from ingested foods, and impaired DAO activity results in an increase in histamine uptake with the corresponding elevation of histamine plasma concentrations [4,5]. Histamine excess may cause a wide range of nonspecific gastrointestinal and extraintestinal symptoms due to the global distribution of histamine receptors in many tissues and organs [5,6,7]. Moreover, DAO enzyme deficiency seems to be the main underlying mechanism of histamine intolerance, which is clinically manifested by a complex combination of digestive, neurological, dermatological, respiratory, and cardiovascular complaints [5,8]. Low levels of DAO have been shown to be a reliable tool in diagnosing histamine intolerance [9,10].

The main cause of DAO enzyme deficiency has a genetic origin. It has been estimated that there is a genetic predisposition in around 80% of cases, with at least one altered variant of the *AOC1* gene. DAO activity is largely associated with single nucleotide polymorphisms (SNPs) of the *AOC1* gene; in particular, the three most relevant variants in Caucasian populations leading to a decrease in DAO enzyme activity are c.47C>T (rs10156191), c.995C>T (rs1049742), and c.1990C>G (rs10449793) [11,12]. In addition, the SNP c.691G>T (rs2052129), which causes a decrease in DAO transcriptional activity, has been identified in the promoter region of the *AOC1* gene [12]. Genotype analyses in genomic DNA obtained from blood samples of 134 Spanish Caucasian individuals showed a frequency of 30.6% (95% confidence interval [CI] 25.1–36.1) for rs10449793, 25.4% (95% CI 20.16–30.58) for rs10156191, and 6.3% (95% CI 3.42–9.26) for rs1049742 [11]. This study also showed that the frequency of individuals with the combination of two variants or carrying together the three variants was between twice and over three times more than expected [11]. These SNPs of the *AOC1* gene should be considered as major determinants of variability for DAO enzyme activity.

Clinical real-world studies have evaluated DAO enzyme deficiency associated with SNPs of the *AOC1* gene in different pathological conditions. In patients with migraines, the DAO SNP rs10156191 showed an odds ratio for the defect allele positivity of 1.61 (95% CI 1.31–2.37) for overall migraine patients and 2.08 (95% CI 1.29–3.36) for women suffering from migraines [13]. In subjects with insomnia-related symptoms, the prevalence of DAO deficiency of a genetic origin was 82.6% [14]. Children and adolescents with attention deficit hyperactivity disorder (ADHD) showed a prevalence of genetic DAO deficiency of 78.5% [15] and women with fibromyalgia of 74.5% [16]. In a cohort of 100 Caucasian adults with symptoms of histamine intolerance, 79% harbored one or more of the four SNPs associated with reduced DAO activity [17]. In a prospective cohort of 100 patients with lower urinary tract symptoms (LUTS), the prevalence of at least one minor allele in the four SNPs of the *AOC1* gene analyzed was 88% [18].

However, up to the present time, the prevalence of DAO enzyme deficiency of a genetic origin in the general population remains undefined. Therefore, the objective of this study was to determine the prevalence of DAO enzyme deficiency defined as having at least one SNP of the *AOC1* gene in a prospective sample of healthy newborns. This approach would allow us to establish the distribution of the four most relevant SNP variants in a general naive asymptomatic population.

## 2. Materials and Methods

### 2.1. Study Design and Population

An observational prospective population-based cohort study of the prevalence of DAO enzyme deficiency in healthy newborns was conducted at Hospital Universitari General de Catalunya, which is a tertiary care teaching hospital in Sant Cugat del Vallés, Barcelona (Spain). Between 30 September 2023 and 31 March 2024, all consecutive newborns who met the eligibility criteria and for which written informed consent was obtained from their parents or tutors were included in the study. Inclusion criteria were as follows: newborns of both genders, born at term (≥37 weeks of gestation). Included newborns had normal findings on physical examination and did not require resuscitation following Apgar score assessment (1 minute after birth or 5 min with a score >7). They were delivered either via cesarean section or vaginal delivery and had appropriate birth weights, without being classified as underweight or macrosomic. It was also necessary that standard ultrasound control examinations and care of the mother had been carried out during pregnancy as well as no abnormalities recorded in the family, maternal, gestational, and perinatal history. Exclusion criteria were prematurity, complications of pregnancy (diabetes, hypertension, infections, preeclampsia, etc.), mothers who had received dietary DAO enzyme supplementation or following a low-histamine diet, and newborns for whom the signed informed consent could not be obtained.

The objective of the study was to determine the prevalence of DAO enzyme deficiency in healthy newborns, which was defined as the presence of at least one of the four most relevant SNP variants of the *AOC1* gene, including c.47C>T (rs10156191), c.995C>T (rs1049742), c.1990C>G (rs10449793), and c.691G>T (rs2052129).

The study was carried out following the principles of the Declaration of Helsinki and Good Clinical Practices (GCP). Personal identification data were maintained confidential during the whole study, and conditions established by General Data Protection Regulation (GDPR) according to the Spanish law were followed. The study protocol was approved by the Clinical Research Ethics Committee (CEIC) of Grupo Hospitalario Quirónsalud-Catalunya (code 2022/95-PED-HUGC, approval date 15 February 2024) and was registered in the ClinicalTrials.gov website (NCT06710366). Written informed consent was obtained from the parents or tutors of all participants (consent forms were written in Spanish).

### 2.2. Study Procedures

Parents or tutors of eligible newborns were personally contacted by the principal investigator (E.F.M.) about 24 h after delivery and were fully informed on the purpose of the study, particularly regarding the non-invasive, painless, and safe method of oral mucosa sampling. They were also told that the final individual report of the genotype with interpretation of the genetic results would be provided.

Genetic variants of the *AOC1* gene were measured in oral mucosa samples obtained from the newborns between 1 and 4 days after birth. Using a sterile cotton swab, study samples were obtained from the inner side of both checks by rubbing. Two samples of buccal mucosa were taken from each patient. Samples were identified using an anonymous code and were maintained at 4 °C for 5 days to preserve the quality of the DNA until analysis. Sample processing and analysis of the *AOC1* genetic SNP variants were performed by Laboratorios Echevarne (Barcelona, Spain). The DNA from oral mucosa samples was obtained with the automated platform M2000SP (Abbott Molecular, Des Plaines, IL, USA). Subsequently, multiplex PCR amplification of the regions of interest of the *AOC1* gene was performed with the Realtype^®^ kit (ref. DDAO-32) (Progenie Molecular SLU, Valencia, Spain) following the manufacturer’s instructions. The SNP variants of the *AOC1* gene analyzed were c.47C>T (rs10156191), c.995C>T (rs1049742), c.1990C>G (rs10449793), and c.691G>T (rs2052129). Results of genomic testing included absence of variants and homozygous and heterozygous status of the alleles of each SNP variant. Genetic DAO enzyme deficiency was defined as the presence of at least one allele variant of the *AOC1* gene.

### 2.3. Statistical Analysis

Descriptive statistics included categorical variables expressed as frequencies and percentages and continuous variables as mean and standard deviation (SD) or median. The 95% confidence intervals (CI) were estimated. The distribution of SNP variants and the number of variants according to gender and ethnicity and comparisons with those of the European population samples in Hardy–Weinberg equilibrium (HWE) extracted from the Allele Frequency Aggregator (ALFA) database [19] were analyzed using either the chi-square test or Fisher’s exact test depending on the sample size and the distribution of data. Statistical significance was set at *p* < 0.05. The R statistical software package (v4.0.0; R Core Team 2020) was used for the analysis of data.

## 3. Results

### 3.1. Study Participants and Prevalence of DAO Deficiency

Over the 6-month study period, the parents or tutors of 200 newborns who met the inclusion criteria accepted to take part in the study and signed the written informed consent. The study population included 96 males (48%) and 104 (52%) females, with a mean (SD) birth weight of 3254 (403) g. The mean gestational age was 41 weeks (SD 2 days). A total of 185 (92.5%) newborns were Caucasian, and the remaining 15 (7.5%) were Latin Americans. The median age at the time of collecting oral mucosa samples was 2 days.

DAO deficiency was present in 132 newborns, with a prevalence rate of 66% (95% CI 59–73%). In 68 newborns (34%), no SNPs were found. The distribution of SNP variants and the number of variants are shown in Table 1. The most frequent variant was c.1990C>G (rs10449793), with a prevalence of 46%, followed by c.47C>T (rs10156191), with a prevalence of 42.5%, and c.691G>T (rs2052129), with a prevalence of 39.5%. The variant c.995C>T (rs1049742) showed the lowest prevalence (9.5%).

### 3.2. AOC1 Genotype and Allelic Variants

The characteristics of allelic variants associated with DAO deficiency are shown in Table 2. In all four SNP variants, heterozygous carriers were more frequent than homozygous carriers. Homozygosity was documented in 19% of the cases.

The comparison of the distribution of the allele and genotype frequencies of the sample with the European population in HEW extracted did not show statistically significant differences (Table 3).

### 3.3. Prevalence of DAO Deficiency by Gender and Ethnicity

Among the 96 male newborns, DAO deficiency was found in 66, with a prevalence rate of 68.8% (95% CI 58.5–77.8), whereas among the 104 female newborns, DAO deficiency was found in 66, with a prevalence rate of 63.5% (95% CI 53.4–72.7). Statistically significant differences in the prevalence of DAO deficiency according to gender were not observed (*p* = 0.523). Also, differences in the number of SNP variants between males and females were not found (*p* = 0.885).

As shown in Table 4, there were no significant differences in the distribution of the four SNP variants between males and females.

In the group of 185 Caucasian newborns, SNPs of the *DAOC1* gene were found in 123 cases, with a prevalence of 66.5%, whereas in the group of 15 Latin Americans newborns, DAO deficiency occurred in 9, with a prevalence of 60% (*p* = 0.821). Differences in the number of SNP variants according to ethnicity were not found either (*p* = 0.568).

## 4. Discussion

The present study carried out in a consecutive population-based sample of healthy newborns shows a high prevalence of DAO enzyme deficiency of a genetic origin of 66%. As far as we are aware, no previous study had evaluated the presence of SNP variants of the *AOC1* gene in newborns. Other important findings include the observation of similar rates in males and females (68.8% vs. 63.4%, respectively) and the fact that DAO deficiency does not seem to be associated with ethnicity, although most participants were Spaniards and only a reduced percentage of 8% were Latin Americans. Another relevant observation was the prevalence of homozygosis of 19%. Of note, previous studies have observed that homozygosis was a predictive factor of a higher occurrence and severity of the symptoms associated with DAO deficiency [15,18].

The genetic study was based on the identification of the alleles of the four variants of the *AOC1* gene most commonly associated with DAO deficiency. In the present study, c.1990C>G (rs1049793) and c.47C>T (rs10156191) accounted for the highest prevalence rates (46% and 42.5%, respectively), and as may be expected, homozygosity was less frequent than heterozygosity.

There is limited evidence on the prevalence of DAO enzyme deficiency of a genetic origin, and a few studies focused on patients with different diseases have been previously published in the literature [13,14,15,16,17,18]. In patients with LUTS, the prevalence of at least one minor allele in the four SNPs analyzed was 88%; in agreement with our study, gender differences were not found, and in all cases, homozygosity with the normal allele was the least prevalent [18]. In 98 Spanish women with fibromyalgia, the prevalence of genetic DAO deficiency was 74.5% based on the four variants of the *AOC1* gene, and the frequency of SNP deficits did not differ from the European population samples extracted from ALFA database [16]. The same findings were observed in our study sample of healthy newborns, in which the frequency of SNPs was similar to that of the European population in the ALFA database [19]. In this study, the c.47C>T (rs10156191) was the most frequent, and 14 genotype combinations among the variants were observed. In the present study, however, combinations of SNP variants were not determined. In a large series of 303 children and adolescents with a mean age of 13.2 years with a primary diagnosis of ADHD and following treatment in a mental health center, the prevalence of having at least one minor dysfunctional allele was 78.8%, with no association between the different variants of the *AOC1* gene and gender [15]. A further interesting piece of data of this study refers to the relationship between severe DAO deficiency due to homozygosity of c.47C>T (rs10156191) and c.691G>T (rs2052129) variants and a lower intellectual quotient and much lower working memory [15]. Finally, in a recent study of the prevalence of DAO deficiency in 167 adult subjects with insomnia-related clinical manifestations, genetic DAO deficiency was found in 82.6% of cases, with trouble staying asleep and early morning awakening as the two symptoms being more frequent in the presence of the c.1990C>G (rs1049793) variant [14].

The high prevalence of genetic predisposition to DAO deficiency found in this study, which seems to be in line with the relevance of DAO enzyme deficiency, is also relevant in the context of other metabolic disorders with a genetic predisposition and a high prevalence in the general population, such as lactose intolerance. Lactose intolerance characterized by lactose malabsorption and irritable bowel syndrome spectrum complaints is a common disorder, with approximately 70% of the human population being lactose intolerant [20]. Loss or deficiency in the lactase enzyme has been associated with SNPs in the *MCM6* regulatory region located upstream of the LCT gene on chromosome 2q21 [21]. Interestingly, it has been shown that low serum DAO values, indicating histamine intolerance, influence the results of a H_2_ lactose breath test in subjects with lactose intolerance. In a study of 402 patients with lactose intolerance, 202 of which had also low DAO values (< 10 U/mL), indicating histamine intolerance, expiratory hydrogen (H_2_) during H_2_ lactose breath tests were significantly higher in patients with combined lactose and histamine intolerance than in patients with lactose intolerance only [22]. Accordingly, in subjects with lactose intolerance, an assessment of SNPs of the *AOC1* gene would be relevant to ascertain a genetic background of DAO enzyme deficiency underlying histamine intolerance.

There is scarce evidence of the frequencies of the variants of the *AOC1* gene in relation to serum levels of DAO enzyme. In the study of Ayuso et al. [11] of 134 healthy Caucasian individuals and in relation to the His645Asp amino acid substitution, it was found that carriers of the variant 645Asp in heterozygosity or homozygosity displayed significantly lower enzyme activity compared with noncarriers. However, in other studies in subjects with migraines [13], fibromyalgia [16], insomnia-related symptoms [14], histamine intolerance [17], ADHD [15], and LUTS [18] in which the prevalence of *AOC1* gene variants was determined, serum levels of DAO enzyme were not measured.

The present findings should be interpreted considering the limitations of the study. The single-center characteristics and the ethnicity of the sample (predominantly Caucasian) could restrict the broader applicability of the findings. In this respect, expanding the study to multiple centers and increasing the sample diversity would provide insights into ethnic variability into SNPs of the *AOC1* gene associated with DAO enzyme deficiency. Serum levels of DAO were not measured, although the need for venipuncture for laboratory analysis could have been a significant drawback in obtaining parental consent. Also, the definition of the healthy status of the babies was based on data collected from the maternal and gestational history and normal findings on physical examination at birth, but extensive work-up studies to assess potential abnormalities were not performed. Combinations of SNP variants were not analyzed, as the objective of the study was not to establish which SNP combinations may be correlated with possible symptoms of DAO enzyme deficiency. In the future, it could be useful to have a registry of children with DAO deficiency of a genetic origin and study which combinations of SNPs could be associated with the symptoms. However, this first real-world study in healthy newborns presents novel evidence of the high prevalence of DAO enzyme deficiency due to SPN variants in the *AOC1* gene in the general population.

## 5. Conclusions

In a consecutive sample of 200 healthy newborns, the prevalence of DAO enzyme deficiency due to alterations in the *AOC1* gene was 66%, without differences according to gender or ethnicity. This high prevalence rate could also serve as a reference value for future investigations. Although these preliminary findings need confirmation in further studies with a larger sample size, they are clinically relevant because they allow for postnatal screening and follow-up to assess the appearance of clinical manifestations of DAO deficiency, particularly histamine-intolerance-related symptoms.

## Figures and Tables

**Table 1:** The prevalence of SNP variants of the *AOC1* gene and the number of SNPs in the study population of 200 healthy newborns.

SNPs of the *AOB1* Gene	Number (%)	95% Confidence Interval
SNPs		
c.1990C>G (rs1049793)	92 (46)	38.9–53.2
c.47C>T (rs10156191)	85 (42.5)	35.6–49.7
c.691G>T (rs2052129)	79 (39.5)	32.7–46.6
c.995C>T (rs1049742)	19 (9.5)	5.8–14.4
Number of SNPs		
One	46 (23)	17.4–29.5
Two	47 (23.5)	17.8–30.0
Three	21 (10.5)	6.6–15.6
Four	18 (9.0)	5.4–13.9

SNP: single nucleotide polymorphism.

**Table 2:** Characteristics of SNP variants of the *AOC1* gene in the study population of 200 healthy newborns.

Genotypes Variants	Number (%)
c.1990C>G (rs1049793) CC	108 (54)
CG	76 (38)
GG	16 (8)
c.47C>T (rs10156191) CC	115 (57.5)
CT	73 (36.5)
TT	12 (6)
c.691G>T (rs2052129) GG	121 (60.5)
GT	70 (35)
TT	9 (4.5)
c.995C>T (rs1049742) CC	181 (90.5)
CT	19 (9.5)

**Table 3:** Allelic and genotype frequencies by the variant for healthy newborns and the European population in Hardy–Weinberg equilibrium.

Samples	Alleles, *n* (%)			Genotype, *n* (%)			
	C	T	*p* Value	CC	CT	TT	*p* Value
p.Thr16Met (rs10156191)							
Newborns (*n* = 200)	303 (75.8)	97 (24.2)	0.408	115 (57.5)	73 (36.5)	12 (6)	0.706
EP-HWE (*n* = 269,740)	199,429 (73.9)	70,311 (26.1)		147,446 (54.7)	103,967 (38.5)	18,327 (6.8)	
p.Ser332Phe (rs1049742)							
Newborns (*n* = 200)	381 (95.2)	19 (4.8)	0.054	181 (90.5)	19 (19.5)	0	0.200
EP-HWE (*n* = 275,948)	255,950 (92.8)	19,998 (7.2)		237,401 (86)	37,098 (13.4)	1449 (0.5)	
p.His664Asp (rs1049793)							
Newborns (*n* = 200)	292 (73.0)	108 (27.0)	0.154	108 (54.0)	76 (38.0)	16 (8.0)	0.310
EP-HWE (*n* = 96,876)	67,537 (69.7)	29,339 (30.3)		47,084 (48.6)	40,907 (42.2)	8885 (9.2)	
c.691G>T (rs2052129)							
Newborns (*n* = 200)	312 (78.0)	88 (22.0)	0.445	121 (60.5)	70 (35.0)	9 (4.5)	0.724
EP-HWE (*n* = 223,202)	170,473 (76.4)	52,729 (23.6)		130,200 (58.3)	80,545 (36.1)	12,457 (5.6)	

EP-HEW: European population in Hardy–Weinberg equilibrium.

**Table 4:** Characteristics of allelic variants of the *AOC1* gene in males and females.

Allelic Variants	Males (*n* = 96) *n* (%)	Females (*n* = 104) *n* (%)	*p* Value
c.1990C>G (rs1049793) CC	53 (55.2)	55 (52.9)	0.909
CG	35 (36.5)	41 (39.4)	
GG	8 (8.3)	8 (7.7)	
c.47C>T (rs10156191) CC	54 (56.2)	61 (58.7)	0.797
CT	37 (38.5)	36 (34.6)	
TT	5 (5.2)	7 (6.7)	
c.691G>T (rs2052129) GG	55 (57.3)	66 (63.5)	0.621
GT	37 (38.5)	33 (31.7)	
TT	4 (4.2)	5 (4.8)	
c.995C>T (rs1049742) CC	87 (90.6)	94 (90.4)	1.00
CT	9 (9.4)	10 (9.6)	

## Data Availability

Data supporting the study findings are available from the corresponding author upon request.
